# Improved method for genomic DNA extraction for *Opuntia* Mill. (Cactaceae)

**DOI:** 10.1186/s13007-017-0234-y

**Published:** 2017-10-11

**Authors:** César Ramiro Martínez-González, Rosario Ramírez-Mendoza, Jaime Jiménez-Ramírez, Clemente Gallegos-Vázquez, Isolda Luna-Vega

**Affiliations:** 1 Laboratorio de Biogeografía y Sistemática, Departamento de Biología, Facultad de Ciencias, Universidad Nacional Autónoma de México, Ciudad Universitaria, Coyoacán, 04510 Mexico City, México; 2 Laboratorio de Biotecnología de Semillas, Colegio de Postgraduados, Carretera México-Texcoco, 56230 Estado de México, México; 3 Herbario de la Facultad de Ciencias, Departamento de Biología Comparada, Facultad de Ciencias, Universidad Nacional Autónoma de México, Ciudad Universitaria, Coyoacán, 04510 Mexico City, México; 4 Centro Regional Universitario Centro Norte, Universidad Autónoma Chapingo, Cruz del Sur núm. 100, Colonia Constelación, El Orito, 98085 Zacatecas, Zacatecas México

**Keywords:** DNA quality, DNA quantity, Genomic DNA, Mucilage, *Opuntia*, Pectin

## Abstract

**Background:**

Genomic DNA extracted from species of Cactaceae is often contaminated with significant amounts of mucilage and pectin. Pectin is one of the main components of cellular walls, whereas mucilage is a complex polysaccharide with a ramified structure. Thus, pectin- and mucilage-free extraction of DNA is a key step for further downstream PCR-based analyses.

**Results:**

We tested our DNA extraction method on cladode tissue (juvenile, adult, and herbaria exemplars) of 17 species of *Opuntia* Mill., which are characterized by a large quantity of pectin and mucilage.

**Conclusion:**

We developed a method for the extraction of gDNA free of inhibitory compounds common in species of *Opuntia* Mill., such as pectin and mucilage. Compared to previously extraction protocols, our method produced higher yields of high-quality genomic DNA.

## Background

Present-day DNA-based molecular studies are useful tools with a wide-range of applications in different biological disciplines. Molecular studies, especially in species with similar morphologies, can be used to characterize and differentiate species [1, 2]. Such studies have used molecular techniques involving PCR amplification of DNA [3, 4] to successfully solve taxonomic and phylogenetic controversies [5]. More specifically, DNA analyses have been used at different taxonomic levels, from communities of bacteria, fungi, yeast, plants and animals, to the cloning of specific genes [6]. High-quality DNA extraction is a necessary first step to conduct molecular studies. This can be performed using conventional methods or commercial kits specifically designed for particular types of samples. Most commercial kits efficiently capture DNA using extraction columns and resins, but the cost of these kits limits their application to large numbers of samples [7].

Conventional methods of DNA extraction involve three basic steps: (1) lysis of cellular walls and membranes; (2) removal of cell debris and other molecular compounds (*e.g.,* polysaccharides, secondary metabolites, proteins, tannins, alkaloids, and polyphenols); (3) DNA precipitation and purification [8]. Currently, fast and cost-efficient DNA extraction protocols yielding large quantities of high-quality DNA are key to the study of species’ molecular genetics [9]. For example, DNA extracted from species of cacti (Cactaceae) are often contaminated with high quantities of mucilage and pectin [10–15].

In these species, pectin is the main component of the cellular wall and its composition often varies among species (*e.g.*, *Opuntia*), location and environments. The main molecular components of pectin are α-(1 → 4) chains linked to d-galacturonic acid interspersed by the insertion of (1 → 2) residues linked to adjacent or alternate residues of l-rhamnopyranosyl. The lineal segments are predominantly composed of homogalacturone [16].

Mucilage is an organic component present in large cells (idioblasts) in the chlorenchyma and adjacent water-retaining parenchymal cells [17, 18]. Mucilage is composed of complex polysaccharides with ramified structures [16] containing varying proportions of different sugars (*e.g.*, l-arabinose, pyranose, furanose, d-galactose, l-rhamnose and d-xylose) and galacturonic acid. The primary structure of the molecule consists of lineal repetitive chains of 1,4-β-d-galacturonic acid and α-1,2-l-rhamnose with a trisaccharide of β-1,6-d-glucose with a lateral chain joined to *O*-4-l-residues of rhamnose [19, 20]. Mucilage is found throughout all body parts, including flowers [11]. In most species of cacti, mucilage is secreted in response to wounds and during the DNA extraction process. More specifically, during the DNA extraction process mucilage appears as soon as the tissue is pulverized, which significantly hinders the efficiency of the extraction and purification [21].

Generally, extraction and purification of high-quality genomic DNA (gDNA) is hindered by the presence of pectin that precipitates alongside DNA [22], thus reducing the quality and yield of the extraction process [23]. Although efficient DNA extraction is crucial for downstream PCR-based analyses, there are relatively few studies focusing on gDNA extraction efficiency in species of cacti [11, 13, 22, 24–27]. In this context, the aim of the present study was to develop a simple and cost-effective method to obtain large yields of high-quality gDNA from cladode tissue of *Opuntia* species.

## Methods

We obtained tissues samples from the national *Opuntia* collection of the Botanical Garden at Instituto de Biología, Universidad Nacional Autónoma de México.

### Protocol

#### CTAB 2X buffer

1. Prepare CTAB 2X buffer solution (Tris 10 mM pH8.0; EDTA 20 mM, pH 8.0; CTAB 2; NaCl 1.4 M) and preheat to 80 °C for 5 min.
2. Pulverize 2–3 mg of tissue using liquid nitrogen.
3. Mix the pulverized tissue with 700 µl of CTAB 2X in a 2 mL eppendorf tube. Mix vigorously for 20 s.
4. Heat to 85 °C for 2 h and mix vigorously for 20 s.
5. Add 750 µl of chloroform: isoamyl alcohol (24: 1) and mix vigorously for 20 s.
6. Centrifuge for 60 min at 12,000 g (4 °C).
7. Transfer the aqueous phase to a 1.5 mL eppendorf tube.
8. Add 400 µl of isopropyl alcohol previously cooled to − 20 °C. Mix gently for 1 min.
9. Centrifuge for 25 min at 10,000 g. Discard the supernatant.
10. Add 500 µl HPLC-grade water to the DNA pellet to dissolve the pectin (evident as a gelatinous substance). Do not mix and discard the disolved pectin with a micropipette.
11. Resuspend the pellet in 1 mL of ethanol (70) previously cooled to − 20 °C.
12. Centrifuge for 5 min at 10,000 g. Discard the supernatant.
13. Air-dry pellet at room temperature for 40 min.
14. Resuspend the pellet in 50 µl of HPLC-grade water.
15. Heat to 60 °C for 15 min.

#### Integrity of the extracted DNA

**Fig. 1:**
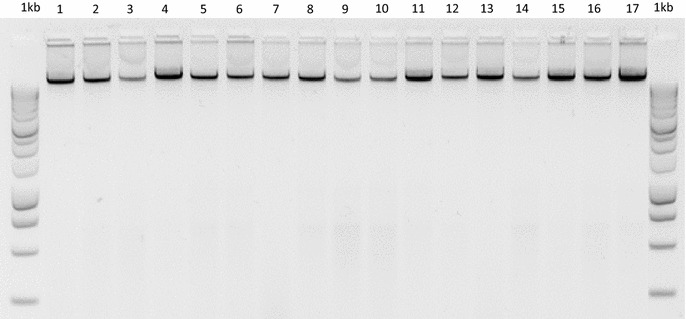
Image of the agarose gel of genomic DNA (gDNA) ran by electrophoresis extracted from 17 tissue samples of *Opuntia* Mill., using the improved extraction method (Promega™ 1 kb DNA Ladder Molecular Weight Marker)

We analyzed the integrity of extracted gDNA from 17 species of *Opuntia* by electrophoresis (1 h with a 87 V cm^−3^ current) using 1.5 agarose gels prepared with TAE buffer (Tris Acetate-EDTA) and stained with Gel red (Biotium, USA). DNA bands were visualized under UV light with an Infinity 3000 transilluminator (Vilber Lourmat, Germany), which confirmed the presence of intact high quality gDNA without conspicuous contamination by proteins or other compounds (Fig. 1).

#### Evaluation of gDNA concentration

We determined gDNA concentration with a spectrophotometry analysis using a NanoDrop 8000 (Thermo, USA) and with a fluorometry analysis using Quant-iT™ PicoGreen^®^ dsDNA Assay Kit (Invitrogen™) according to the manufacturer’s instructions.

#### Evaluation of the quality of gDNA

We assessed the purity of all the gDNA samples by spectrophotometry with a Nanodrop 8000 (Thermo, USA) (Table 1).

**Table 1:** Genomic DNA (gDNA) concentration and quality extracted from 17 tissue samples of *Opuntia* Mill. using the improved extraction method

Species	PicoGreen ng/µl	NanoDrop ng/µl	C B ratio PicoGreen concentration/Nanodrop concentration	A_260_/A_280_ NanoDrop	A_260_/A_230_ NanoDrop
1. *Opuntia auberi* Pfeiff.	1250	1500	0.83	1.9	2.1
2. *Opuntia decumbens* Salm-Dyck	3199	3642	0.87	1.9	2.2
3. *Opuntia delafuentiana* Martínez-González et al.	8021	8126	0.98	1.9	2.2
4. *Opuntia depressa* Britton and Rose	2191	2588	0.84	1.9	2.0
5. *Opuntia durangensis* Britton and Rose	8220	8853	0.92	1.8	2.1
6. *Opuntia ficus*-*indica* Mill.	5898	6196	0.95	1.9	2.1
7. *Opuntia heliabravoana* Scheinvar	8341	9147	0.91	2.0	1.9
8. *Opuntia huajuapensis* Bravo	3624	4497	0.80	1.9	2.1
9. *Opuntia joconostle* F. A. C. Weber	1091	1304	0.83	1.8	2.2
10. *Opuntia lasiacantha* Pfeiff.	1892	2088	0.90	1.9	2.1
11. *Opuntia leiascheinvariana* Martínez-González	4799	5407	0.88	1.9	2.2
12. *Opuntia leucotricha* DC.	6258	7000	0.89	1.9	2.2
13. *Opuntia matudae* Scheinvar	2354	2802	0.84	1.9	2.2
14. *Opuntia megacantha* Salm-Dyck	6895	7861	0.87	1.8	2.1
15. *Opuntia microdasys* Pfeiff.	7526	8592	0.87	1.9	2.1
16. *Opuntia oligacantha* Förster	1548	1897	0.81	1.8	2.2
17. *Opuntia olmeca* Joel Pérez et al.	2112	2568	0.82	1.9	2.1

#### PCR amplifications

The purity of gDNA was confirmed through PCR of three different molecular markers: (1) nDNA internal transcribed spacer (ITS, 600 bp) [28–32]; (2) cpDNA RuBisCO gene (*rbc*L, 500 pb) [33, 34]; (3) mtDNA cytrochrome oxidase subunit 3 (*cox*3, 1000pb) [35]. We used a negative control (without target gDNA) to confirm no contamination with extraneous DNA before the PCR. PCRs were performed on a final volume 25 µL containing 1 × buffer, 0.8 mM dNTPs mix, 20 pmol of each primer, 2 units of Go*Taq* DNA (Promega, USA) and 100 ng of template DNA. For each gene, PCRs consisted of an initial denaturation step at 96 °C for 2 min, followed by 35 cycles at 94 °C for 1 min, annealing temperature differing according to the primer for 1 min (Table 2), 72 °C elongation temperature for different time durations, depending on the length of the product. PCRs were performed using a Peltier Thermal Cycler PTC-200 (BIORAD, México). Amplification products were subjected to electrophoresis (1 h with a 87 V cm^−3^ current) using 1.5 agarose gels prepared with TAE buffer (Tris Acetate-EDTA), stained with Gel red (Biotium, USA) and visualized with an Infinity 3000 transilluminator (Vilber Lourmat, Germany). PCR products were purified with the ExoSAP Purification kit (Affymetrix, USA) and sequenced using the Bigdye terminator v.3.1 Cycle Sequencing kit (Applied Biosystem) and an Applied Biosystems 3730 × L automated sequencer (Applied BioSystems, USA).

**Table 2:** Primers used in the amplification and sequencing of the DNA fragments

Locus/segment	Name	Sequence 5′–3′	Tm (°C)
ITS	ITS5	GGAAGTAAAAGTCGTAACAAGG	57
	ITS4	TCCTCCGCTTATTGATATGC	57
*rbc*L	1f	ATGTCACCACAAACAGAAAC	56
	724r	TCGCATGTACCTGCAGTAGC	56
*cox*3	Cox3f	CCGTAGGAGGTGTGATGT	51
	Cox3r	CTCCCCACCAATAGATAGAG	51

#### Sequence assembly

DNA sequences were visualized, edited and assembled using BioEdit vers. 7.0.5 [36]. For each gene, consensus sequences were compared with those deposited in GenBank using the BLASTN 2.2.19 search algorithm [37].

#### Comparison with previous methods

Our protocol was compared with two previous methods [11, 13] using 17 species of *Opuntia*.

Only one species (*Opuntia ficus*-*indica*) was shared with the protocol of Mondragón et al. [11].

## Results

The list of the 17 species of *Opuntia* studied is shown in Table 1.

Our new extraction method allowed us to obtain high quality gDNA from young and mature cladodes using standard protocols using CTAB (Cetyl Trimethyl Ammonium Bromide), which efficiently extracts polysaccharides from leaf tissue. The Agarose gel electrophoresis showed the presence of large quantities of gDNA free of contaminants (Fig. 1). Accordingly, the large amount of gDNA was confirmed with two different methods (*i.e.*, spectrophotometry and fluorimetry). These analyses yielded a mean gDNA ratio (PicoGreen concentration/Nanodrop concentration) of 0.80–0.98 ng/µl for all of the samples tested (Table 1). We obtained reliable absorbance readings from the spectrophotometric analysis.

The estimation of the A_260_/A_280_ absorbance ratio is a common way to measure DNA purity. Nucleic acids have a maximum absorbance at a wavelength of 260 nm, thus absorbance at this wavelength is directly proportional to DNA concentration. On the other hand, proteins show a maximum absorbance at 280 nm wavelength (mainly resulting from tryptophan residues), thus absorbance readings at 280 nm measure the concentration of proteins in the sample. Depending on the base composition of DNA, reading for the A_260_/A_280_ ratio between 1.6 and 1.9 are indicative of high-quality DNA. In addition, absorbance readings at 230 nm wavelength measure the concentration of salts, carbohydrates and other contaminants, so the A_260_/A_230_ absorbance ratio should also be considered. Both A_260_/A_280_ and A_260_/A_230_ absorbance ratios are typically used to determine the purity of DNA samples that were extracted using biological, organic and inorganic compounds. Sambrook et al. [8] suggested that when measuring pure double-stranded DNA, the A_260_/A_280_ and A_260_/A_230_ absorbance ratios should ideally be in the range of 1.6–1.9 and 2.0–2.2, respectively. Accordingly, our absorbance analysis for all samples yielded values for A_260_/A_280_ and A_260_/A_230_ within the ideal range (Table 1), which is indicative of high quality of the extracted gDNA.

**Fig. 2:**
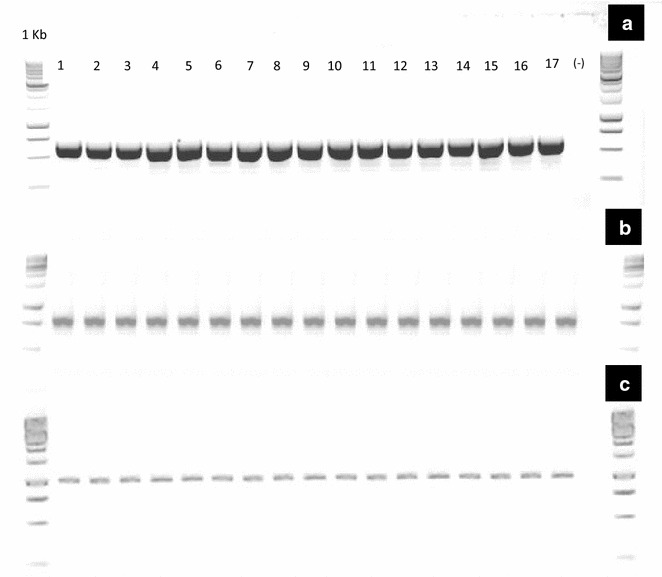
Image of the agarose gel of the PCR products (gDNA) ran by electrophoresis obtained from genomic DNA (gDNA) extracted from 17 tissue samples of *Opuntia* Mill., using the improved extraction method. **a** nDNA internal transcribed spacer (ITS), **b** cpDNA RuBisCO gene (*rbc*L), **c** mtDNA cytochrome oxidase subunit 3 (*cox*3) (Promega™ 1 kb DNA Ladder Molecular Weight Marker)

**Fig. 3:**
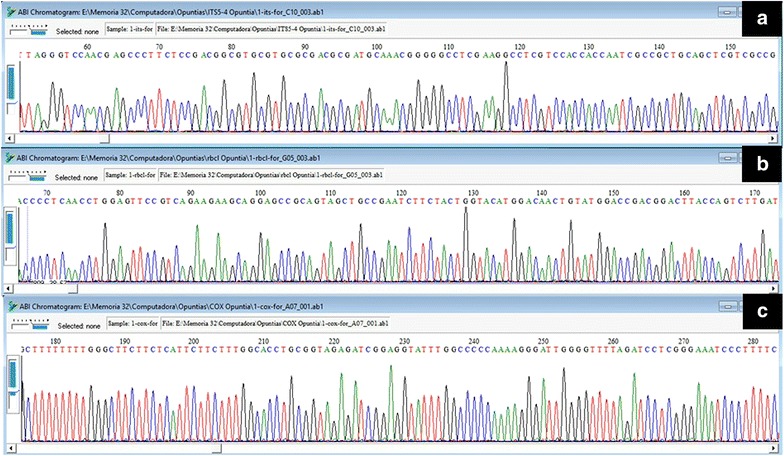
DNA sequence chromatograms for PCR products obtained from genomic DNA (gDNA) samples extracted from 17 tissue samples of *Opuntia* Mill using the improved extraction method. **a** nDNA internal transcribed spacer (ITS), **b** cpDNA RuBisCO gene (*rbc*L), **c** mtDNA cytochrome oxidase subunit 3 (*cox*3). Sequences were visualized using BioEdit v 7.0.5

PCRs of rbc*L*, *cox*3 and ITS regions were successful for all samples (Fig. 2). DNA sequencing for all three regions was successful (Fig. 3), which allowed us to construct high-quality consensus sequences for all three regions.

In order to complement sequence quality assessment, we decided to assess the identity of sequences, at least preliminary, with a basic BLAST search. It has been documented that BLAST is not the proper mean for taxonomical identification, but it provides an easy way to broadly verify if the sequence belongs to the sample (*e.g.*, verifying a potential contamination).

We conducted a BLAST search for each of the 17 sequences and the first hit on each search was recorded (Table 3). All the searches hit in sequences of *Opuntia*, but only five ITS sequences matched with the corresponding species. The other loci (*rbc*L and *cox*3) matched on *Opuntia* as well, but with non-corresponding species.

**Table 3:** Blast search for the three markers

Species number	Description	Max score	Total score	Query cover (%)	E value	Ident (%)	Accession
ITS							
1	*Opuntia* sp.	865	865	100	0.0	100	JF787077.1
2	*Opuntia bravoana*	929	929	100	0.0	100	JF87044.1
3	*Opuntia delafuentiana*	968	968	100	0.0	100	KM67822.1
4	*Opuntia depressa*	822	822	100	0.0	99	JF787089.1
5	*Opuntia martiniana*	963	963	100	0.0	100	JF787066.1
6	*Opuntia ficus*-*indica*	1059	1059	100	0.0	100	JF78710.1
7	*Opuntia robusta*	1048	1048	100	0.0	99	JF787122.1
8	*Opuntia velutina*	850	850	100	0.0	100	HQ872589.1
9	*Opuntia martiniana*	1094	1094	100	0.0	100	JF787066.1
10	*Opuntia pittieri*	1109	1109	100	0.0	100	JF787105.1
11	*Opuntia leiascheinvariana*	970	970	100	0.0	100	KM507353.1
12	*Opuntia cubensis*	1027	1027	100	0.0	100	JF787058.1
13	*Opuntia martiniana*	1003	1003	100	0.0	100	JF787066.1
14	*Opuntia pittieri*	1120	1120	100	0.0	100	JF787105.1
15	*Opuntia carstenii*	992	992	100	0.0	100	JF787112.1
16	*Opuntia oligacantha*	953	953	100	0.0	100	KX247005.1
17	*Opuntia bakeri*	1059	1059	100	0.0	99	JF787101.1
*rbc*L							
1	*Opuntia maxima*	1245	1245	100	0.0	100	HM850212.1
2	*Opuntia dillenii*	1262	1262	99	0.0	100	HM850211.1
3	*Opuntia maxima*	1254	1254	100	0.0	100	HM850212.1
4	*Opuntia maxima*	1262	1262	99	0.0	100	HM850212.1
5	*Opuntia maxima*	1258	1258	99	0.0	100	HM850212.1
6	*Opuntia dillenii*	1258	1258	99	0.0	100	HM850211.1
7	*Opuntia dillenii*	1262	1262	99	0.0	100	HM850211.1
8	*Opuntia maxima*	1260	1260	99	0.0	100	HM850212.1
9	*Opuntia maxima*	1262	1262	99	0.0	100	HM850212.1
10	*Opuntia maxima*	1260	1260	99	0.0	100	HM850212.1
11	*Opuntia maxima*	1260	1260	99	0.0	100	HM850211.1
12	*Opuntia maxima*	1260	1260	99	0.0	100	HM850212.1
13	*Opuntia maxima*	1253	1253	99	0.0	100	HM850212.1
14	*Opuntia maxima*	1090	1090	100	0.0	100	HM850212.1
15	*Opuntia dillenii*	1262	1262	99	0.0	100	HM850211.1
16	*Opuntia maxima*	1085	1085	100	0.0	100	HM850212.1
17	*Opuntia maxima*	1254	1254	99	0.0	100	HM850212.1
*cox*3							
1	*Opuntia megacantha*	1117	1117	100	0.0	100	EU930402.1
2	*Opuntia megacantha*	1033	1033	100	0.0	100	EU930402.1
3	*Opuntia megacantha*	1125	1125	100	0.0	100	EU930402.1
4	*Opuntia megacantha*	900	900	100	0.0	100	EU930402.1
5	*Opuntia megacantha*	1212	1212	100	0.0	100	EU930402.1
6	*Opuntia albicarpa*	1179	1179	100	0.0	100	EU930396.1
7	*Opuntia megacantha*	1249	1249	100	0.0	100	EU930402.1
8	*Opuntia megacantha*	1175	1175	100	0.0	100	EU930402.1
9	*Opuntia megacantha*	1236	1236	100	0.0	100	EU930402.1
10	*Opuntia megacantha*	1234	1234	100	0.0	100	EU930402.1
11	*Opuntia megacantha*	1201	1201	100	0.0	100	EU930402.1
12	*Opuntia megacantha*	1223	1223	100	0.0	100	EU930402.1
13	*Opuntia matudae*	1225	1225	100	0.0	100	EU930401.1
14	*Opuntia megacantha*	1238	1238	100	0.0	100	EU930402.1
15	*Opuntia megacantha*	1171	1171	100	0.0	100	EU930388.1
16	*Opuntia megacantha*	1173	1173	100	0.0	100	EU930402.1
17	*Opuntia megacantha*	985	985	100	0.0	100	EU930402.1

In this table is only recorded the first hit on each search

BLAST results on *rbc*L and *cox*3 are due to the fact that those loci have very low variability at species level. Sequence variability was not enough for proper species identity, but sufficient for genera identity.

On the other hand, ITS is a loci with larger variability at species level. We found five searches that matched with the corresponding species. At four searches, the corresponding species were not available in GenBank, and no correct match was possible, but the search hit in *Opuntia.* The remaining searches on the ITS sequences did not match on the correct species, but did match in *Opuntia*. This result is due to two main reasons: 1) the BLAST search is not designed for species match, even if the species are available in the database, and in consequence it is not a suitable tool for specimens identification; and 2) because in most cases our sequences are longer (including ITS1 and 2 as well as 5.8S region) than those available in GenBank; this extra length may induce some errors.

### Comparison with previous methods

We replicated the protocols of Mondragón-Jacobo et al. [11] and Griffith and Porter [13] using the same 17 species of *Opuntia* (Table 4). We confirmed that our method got better performance (quality and quantity of gDNA), and that it has some advantages over other protocols (Table 5). In addition, our protocol is the cheapest one and considered as a micro-method due to the amounts of reagents and tissue involved.

**Table 4:** Comparison among three different protocols to obtain total genomic DNA using NanoDrop

Species	Mondragón-Jacobo et al. [11]		Griffith and Porter [13]		This protocol	
	DNA yield (ng/µl)	OD ratio _260.280_	DNA yield (ng/µl)	OD ratio _260.280_	DNA yield (ng/µl)	OD ratio _260.280_
1. *Opuntia auberi* Pfeiff.	256	1.4	423	1.7	1600	1.9
2. *Opuntia decumbens* Salm-Dyck	35	1.7	30	1.9	2930	1.9
3. *Opuntia delafuentiana* Martínez-González et al.	75	1.6	56	1.9	4937	1.8
4. *Opuntia depressa* Britton and Tose	95	1.9	73	1.8	8755	1.9
5. *Opuntia durangensis* Britton and Tose	134	1.5	123	1.7	5835	1.9
6. *Opuntia ficus*-*indica* Mill.	34	1.8	258	1.8	3829	2.0
7. *Opuntia heliabravoana* Scheinvar	198	1.6	43	1.8	8743	1.8
8. *Opuntia huajuapensis* Bravo	57	1.5	78	1.7	1573	1.9
9. *Opuntia joconostle* F.A.C. Weber	86	1.9	196	1.0	8375	1.8
10. *Opuntia lasiacantha* Pfeiff.	67	1.7	356	1.7	2943	1.9
11. *Opuntia leiascheinvariana* Martínez-González	110	1.8	98	1.9	3980	1.9
12. *Opuntia leucotricha* DC.	248	1.5	34	1.8	3789	1.9
13. *Opuntia matudae* Scheinvar	93	1.7	63	1.8	7947	1.9
14. *Opuntia megacantha* Salm-Dyck	117	1.6	78	1.8	7000	1.8
15. *Opuntia microdasys* Pfeiff.	44	1.5	39	1.7	6578	1.9
16. *Opuntia oligacantha* Förster	87	1.8	70	1.9	2395	1.8
17. *Opuntia olmeca* Joel Pérez et al.	94	1.5	57	1.7	9200	1.9

**Table 5:** Advantages of our protocol

Mondragón-Jacobo et al. [11]	Griffith and Porter [13]	This protocol
They tried to use young tissues, avoiding older ones because their higher content of fiber and cuticular wax	They tried to use epidermal tissue free of waxes	We can use tissue from any part of the plant
They used β-mercaptoethanol	They used β-mercaptoethanol	We did not use β-mercaptoethanol
8000 mg of cactus pear tissue	30–50 mg of epidermal tissue	2–3 mg of tissue from every part of the plant
They used more CTAB (25 ml)	They used more CTAB (15 ml)	We used few CTAB (0.7 ml)
They used more chloroform-isoamyl alcohol (10 ml)	They used more chloroform-isoamyl alcohol (5 ml)	We used few chloroform-isoamyl alcohol (0.75 ml)
They used ethanol (8.7 ml)	They used more isopropanol (5 ml)	We used few isopropanol (0.4 ml)
They used bigger and expensive tubes (15 ml)	They used bigger and expensive tubes (15 ml)	We used smaller tubes (2 ml)
They used RNAse to eliminate RNA	They did not use RNAse	We did not use RNAse

## Discussion

Several gDNA extraction protocols were developed recently, but few of these have been focused on the elimination of pectin and polysaccharides. These two compounds are among the most difficult contaminants to separate from the DNA [38] and significantly interfere with the activity of DNA polymerases. Therefore, the elimination of these compounds during the extraction of gDNA favors the efficiency of PCR amplification [39]. Pectin and mucilage (polysaccharides) are two of the main tissue components tissue in *Opuntia*. More specifically, pectin is the main component of the middle layer of cell walls and mucilage is one of the principal components of the parenchyma.

Mondragón-Jacobo et al. [11] developed a DNA extraction method for several cacti species (*e.g.*, *Cleistocactus* spp., *Echinocereus* spp., *Nopalea* spp., *Opuntia* spp., *Stenocereus* spp.). The amount of tissue used in this extraction protocol is species-dependent due to varying mucilage content among species. Griffith and Porter [13] extracted DNA from epidermal cells from several species of *Austrocylindropuntia*, *Brasilopuntia*, *Consolea*, *Cumulopuntia*, *Cylindropuntia*, *Grusonia*, *Maihueniopsis*, *Miqueliopuntia*, *Nopalea*, *Opuntia*, *Pereskiopsis*, *Pterocactus*, *Tephrocactus* and *Tunilla*. In recent years, Mihalte et al. [25] showed that the protocol of Pop et al. [30] yielded sufficient amounts of DNA from small amounts of tissue for species of *Rebutia*, *Mediolobivia*, *Sulcorebutia* and *Aylostera*. Accordingly, Yu et al. [26] introduced a protocol, similar to that of Pop et al. [30], for reliable DNA extraction from *Hylocereus* spp. Montiel et al. [27] used root tissue from *Opuntia* to extract DNA due to the difficulties encountered during extraction from cladode tissue. Wong et al. [22] developed a method to extract DNA from *Hylocereus* spp. Out of these studies, only those of De la Cruz et al. [10], Mondragón-Jacobo et al. [11], Griffith and Porter [13], Montiel et al. [27] and Fehlberg et al. [40] tested extraction efficiency on species of *Opuntia*.

Our improved gDNA extraction method is based on the protocols of Mondragón-Jacobo et al. [11] and Griffith and Porter [13]. We developed this method for the extraction of DNA from *Opuntia* cladodes, which contain large quantities of mucilage and pectin [20]. More specifically, improvements in the method involved changes to centrifugation and incubation steps (*e.g.*, increased times and temperatures), the addition of water to remove pectin and the elimination of various reactive agents, such as polyvinylpyrrolidone (PVP), β-mercaptoethanol and protein and RNA degrading enzymes.

The increased centrifugation times allowed for a better separation of gDNA from fiber cells and non-soluble cellular components, such as proteins. As pectin is water-soluble, the addition of water permitted the extraction of this compound, forming a gelatinous substance over the precipitated gDNA [41, 42].

Generally, polyvinylpyrrolidone (PVP) is used to suppress polyphenolic oxidation during the extraction process [43]. However, PVP was not used because the main issue associated with DNA extraction from *Opuntia* samples is the presence of pectin and mucilage, and not of phenolic compounds.

The longer time of incubation at higher temperatures results in a more efficient denaturation of the proteins and enzymes found in tissue samples of *Opuntia*. Therefore, the extra step of incubation with proteinases is not needed.

The Β-mercaptoethanol inhibits the activity of DNAs and RNAs and thus protects gDNA from degradation. However, we do not use this compound in our extraction protocol because EDTA (contained in CTAB) forms a molecular complex with Mg2 + ions that prevents the functioning of DNAs [8]. In turn, we do not use RNAse because we included a final drying step for 40 min, followed by 15 min at 60 °C, that allows for the efficient degradation of RNA.

Ribonucleases (RNAses) are abundant in all biological and most of these are fairly stable and difficult to inactivate even when extraction reagents and materials have been autoclaved. Thus, when extracting RNA from biological samples RNAses should be eliminated rapidly with denaturing compounds [8]. The presence of RNA in the samples is controlled with the fluorimetry analysis using the Quant-iT ™ PicoGreen^®^ Kit (Invitrogen™), which is an ultra-sensitive method for quantifying double-stranded DNA. The determination of absorbance at 260 nm wavelength is the commonly used technique for measuring the overall concentration of nucleic acids. However, absorbance measures have the main disadvantage of confounding the absorbance contribution of single-stranded nucleic acids, thus being unable to distinguish between DNA and RNA.

The purity of the extracted gDNA was confirmed by spectrophotometry. Generally, a higher A_260_/A_280_ value is indicative of RNA contamination, whereas lower values are indicative of protein contamination. On the other hand, lower A_260_/A_230_ values indicate the presence of phenolic compounds and carbohydrates, whereas higher values are usually associated with calibration errors [44]. The A_260_/A_280_ and A_260_/A_230_ ratios for dsDNA ideally range from 1.6 to 1.9 and from 2.0 to 2.2, respectively [8]. Our analyses showed A_260_/A_280_ and A_260_/A_230_ within these ideal ranges (Table 1), which confirm the purity of the gDNA samples. Through the improvement of DNA extraction protocols, we were able to improve the overall yield and purity of gDNA (1500–9147 ng/μl, Table 1) extracted from different species of *Opuntia*. In addition, with these changes, the extraction protocol becomes cheaper and the use of toxic reagents is diminished.

When we compared our method with other two previous protocols [11, 13], we observed that the necessary amount of tissue in these two protocols is huge. Also, both methods need a great amount of expensive chemical reagents, making them impractical. With our new protocol, we obtained a higher DNA performance with high molecular weight (1500 ng/μl), and an average of the ratio A_260_/A_280_ of 1.8.

Our protocol is a good alternative to these methods, since it requires milligrams of tissue and small volumes of reagents, facilitating the handling of a large number of samples. In short, our method is cheaper, quick and simple, and it does not need to carry out additional purification.

## Conclusion

In this study, we developed a method of DNA extraction that yields high-quality gDNA free of inhibitory organic compounds common in species of *Opuntia*, such as pectin and mucilage. This improved method allowed us to obtained higher yields of gDNA of excellent quality. Our method works in other species of cacti (*e.g.*, *Nopalxochia* [45]); it will be interesting to test it in other Cactaceae and succulent plants such as Crassulaceae. Finally, we are demonstrating that the addition of RNAses is not necessary to remove RNA from the genomic DNA samples. The use of RNAse is replaced by a heat treatment to remove the RNA making the protocol cheaper.
